# Effect of histone H4 tail on nucleosome stability and internucleosomal interactions

**DOI:** 10.1038/s41598-021-03561-9

**Published:** 2021-12-16

**Authors:** Tommy Stormberg, Sridhar Vemulapalli, Shaun Filliaux, Yuri L. Lyubchenko

**Affiliations:** Department of Pharmaceutical Sciences, University of Nebraska Medical Center, Omaha, NE 68198-6025 USA

**Keywords:** Biochemistry, Biophysics, Molecular biology, Structural biology, Atomic force microscopy

## Abstract

Chromatin structure is dictated by nucleosome assembly and internucleosomal interactions. The tight wrapping of nucleosomes inhibits gene expression, but modifications to histone tails modulate chromatin structure, allowing for proper genetic function. The histone H4 tail is thought to play a large role in regulating chromatin structure. Here we investigated the structure of nucleosomes assembled with a tail-truncated H4 histone using Atomic Force Microscopy. We assembled tail-truncated H4 nucleosomes on DNA templates allowing for the assembly of mononucleosomes or dinucleosomes. Mononucleosomes assembled on nonspecific DNA led to decreased DNA wrapping efficiency. This effect is less pronounced for nucleosomes assembled on positioning motifs. Dinucleosome studies resulted in the discovery of two effects- truncation of the H4 tail does not diminish the preferential positioning observed in full-length nucleosomes, and internucleosomal interaction eliminates the DNA unwrapping effect. These findings provide insight on the role of histone H4 in chromatin structure and stability.

## Introduction

In eukaryotes, genetic DNA must be packaged into chromatin to fit inside the nucleus. Nucleosomes, the fundamental repeating units of chromatin, tightly wrap ~ 147 bp of DNA each and are separated by ~ 20–90 bp of linker DNA to form higher order structures and achieve the compact chromatin structure^1–3^. Genetic DNA must be tightly compacted, but easily accessible for gene transcription. Nucleosomes, however, can act as a barrier to transcription factors and inhibit the transcription of wrapped DNA^4–6^. One of the ways this barrier is overcome is by post-translational modifications (PTMs) to the histone tails of nucleosomes. Histone tails are known to play a critical role in chromatin structure, and PTMs are used to regulate their role^7–9^. Histone H4 acetylation, for example, is necessary for opening chromatin during replication^10^, whereas histone H4 methylation can regulate DNA replication by compacting chromatin^11^. Regulation of chromatin structure is critical, as dysregulation of these systems are observed in many diseases, including cancer^12–14^.

The nucleosome core is made up of two copies each of histones H2A, H2B, H3, and H4^15^. Internucleosomal interactions between histone tails play a large role in the stability of chromatin structure, even at a distance^16^. The histone H4 tail in particular is involved in these internucleosomal interactions^17–19^, as well as in DNA break repair^20,21^. Despite the role of histone H4 in these interactions, it has been reported that complete truncation of the histone tail does not significantly affect nucleosome structure; however, these conclusions were reported on mononucleosome results of simulations^22^ and assembled on positioning sequences^23^. The effect of H4 tail truncation on higher order nucleosomes and nucleosomes assembled on nonspecific DNA is not well understood due to the heterogeneous nature of nucleosome samples not assembled on positioning sequences and the lack of precise positioning tools used to analyze such assemblies.

Here we utilize Atomic Force Microscopy (AFM) with the capability to characterize nucleosomes on the single molecule level with nanometer resolution to overcome these difficulties^24–26^. We assembled canonical and H4 tail truncated mono and dinucleosomes on the strong positioning 601 and nonspecific DNA substrates. Canonical dinucleosomes were assembled on the positioning substrates for comparison. We found that H4 tails contribute to the DNA wrapping and internucleosomal interactions. The implications of these data on nucleosome structure and internucleosomal interaction are discussed.

## Results

### AFM analysis of H4 truncated mononucleosomes

H4 truncated nucleosomes were assembled on the mononucleosome substrates shown in Fig. S1A, B. Substrate in (A) contains the specific 601 sequence, whereas substrate in (B) has the mixed non-specific sequence (MS) of the same length, which did not contain any nucleosome specific motifs. Both substrates are 377 bp long with arm flanks of 113 and 117 bp. On the MS substrate, the 147 bp of the centrally positioned 601 sequence is replaced by 147 bp of MS DNA. DNA were terminated with biotin on the 117 bp flank allowing for distinction between the left and right ends on assembled nucleosomes after labeling with streptavidin or rhizavidin. The end labeling was done before the deposition of the nucleosome samples for AFM imaging.

**Figure 1:**
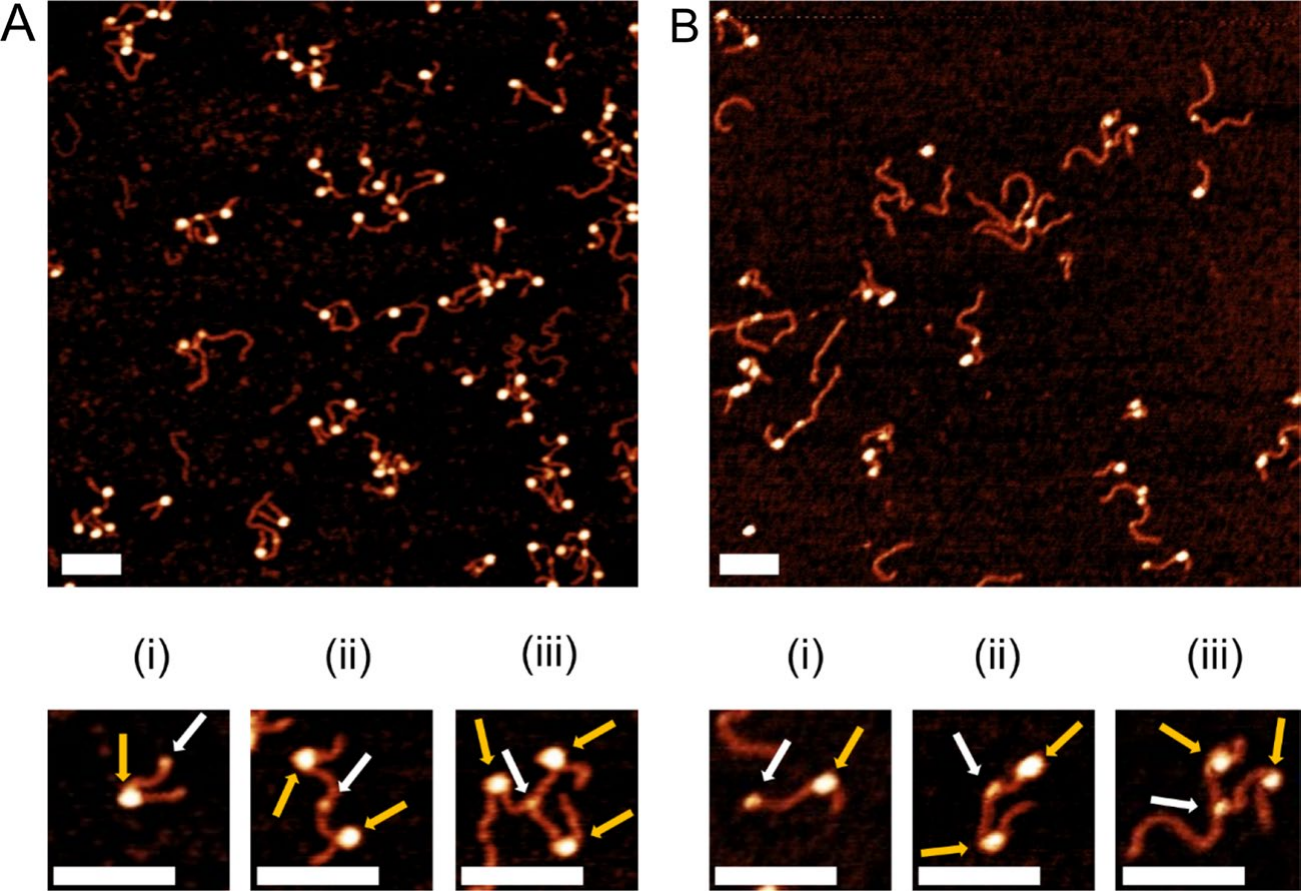
Representative images of mononucleosome samples with snapshots below. White arrows indicate streptavidin and gold arrows indicate the nucleosome core. Scale bars indicate 100 nm. (i), (ii), and (iii) show one, two, and three nucleosomes bound by streptavidin, respectively. (**A**) Nucleosomes assembled on 601 substrate. (**B**) Nucleosomes assembled on MS substrate.

Images of the samples assembled on the histone cores with truncated H4 are shown in Fig. 1. A representative image of nucleosomes assembled on the 601 substrate is shown in Fig. 1A,B shows a representative image of nucleosomes assembled on the MS substrate. Selected zoomed images of typical mononucleosome samples are shown in plates (i) for both samples in which the nucleosome and streptavidin are indicated. Gold arrows indicate the nucleosome core and white arrows indicate streptavidin. Streptavidin is capable of binding several biotinylated DNA molecules, producing the assemblies shown in plates (ii) and (iii). From these images, we were able to obtain descriptive parameters such as flank length, nucleosome position, and wrapping efficiency as described in the Methods section.

**Figure 2:**
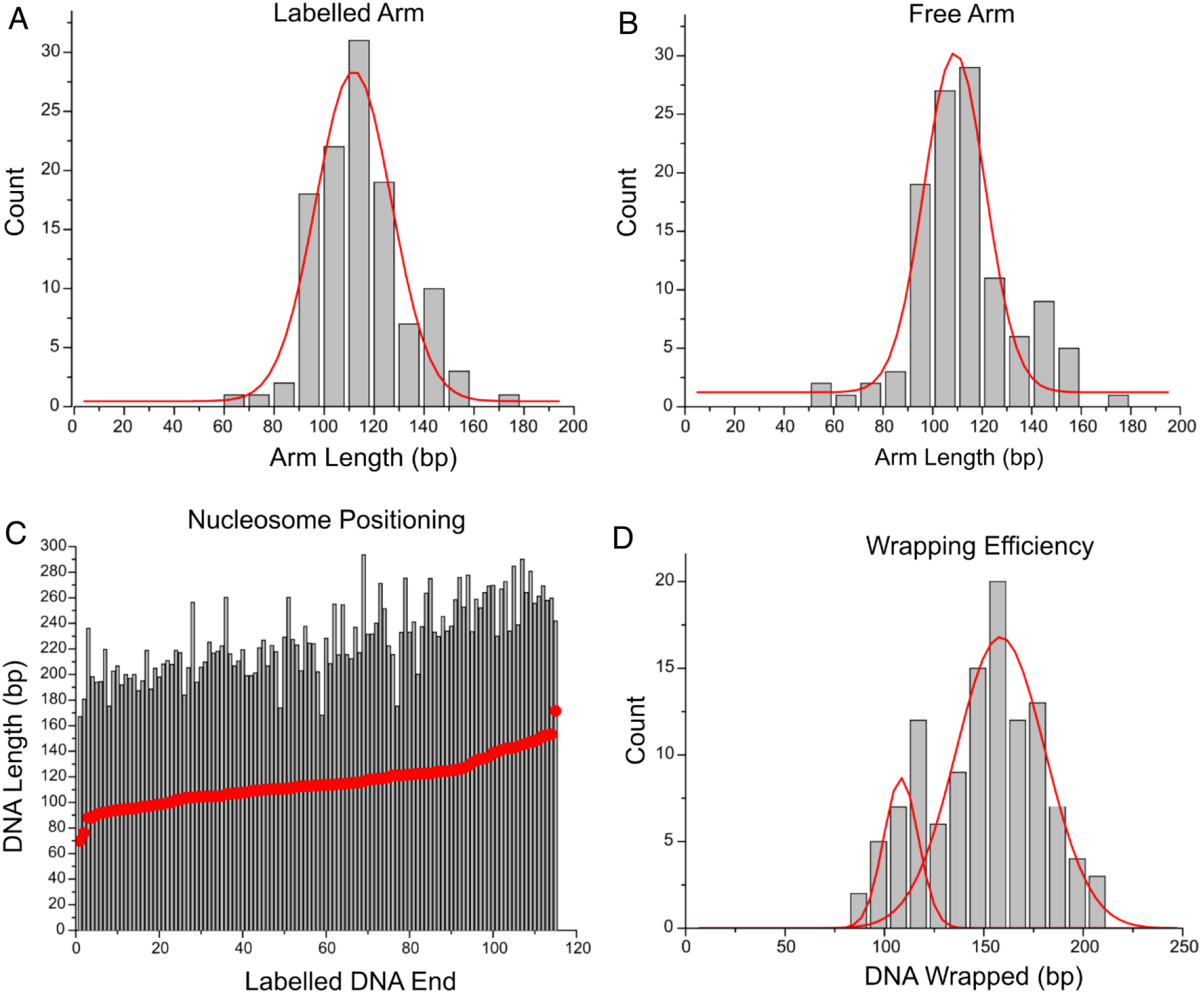
Analysis of modified mononucleosomes with the H4 histone tail truncated assembled on the 601 positioning substrate. Histograms (**A**,**B**) reveal labelled and free arm lengths of 116 ± 2 bp (SEM) and 113 ± 2 bp (SEM), respectively. (**C**) Positional mapping of modified nucleosomes on the positioning substrate demonstrates positioning according to the location of the 601 motif. (**D**) Histogram of nucleosome wrapping efficiency. The mean value of wrapping efficiency is 147 ± 3 bp (SEM). Under and over wrapped populations are fitted by Gaussian function.

To characterize the specificity of the nucleosome location, the lengths of both flanks were measured. The flank lengths were determined by the measuring distances from the streptavidin-bound end and the free end to the center of the nucleosome and subtracting 5 nm from each flank to account for the contribution of the nucleosome core diameter. The data for nucleosomes assembled on the 601 substrate are shown in in Fig. 2A,B. The histograms show narrow peaks with mean values of 116 ± 2 and 113 ± 2 bp (SEM) for the labeled and unlabeled arms, respectively. These values are consistent with the nucleosome position at the 601 motif (Fig. S1A).

Next, we mapped the nucleosome positions on the DNA template as done previously^26^. The maps are shown in Fig. 2C. Each bar represents a single nucleosome complex. The red dots indicate the position the nucleosome begins wrapping DNA with the zero position corresponding to the streptavidin-bound end of the DNA substrate. Maps are organized by distance from the streptavidin bound DNA end for each nucleosome complex from nearest to furthest. The 601-positioning sequence begins at 117 bp from the streptavidin-bound DNA terminus and ends 113 bp from the free DNA end. We found that nucleosomes are narrowly positioned within the Widom 601 region, indicating that the specificity of the DNA sequence for nucleosome positioning is not hindered by the truncation of the H4 histone tail.

We also measured the nucleosome wrapping efficiency, and the results are shown in Fig. 2D. The histogram was generated by subtracting the measured flank lengths from the total length of the DNA substrate (377 bp). Gaussian fits of the histogram demonstrated that H4 truncated nucleosomes display a bimodal distribution, with a large broader peak corresponding to a slightly over wrapped conformation of nucleosomes and another peak with corresponding to a minor unwrapped nucleosome fraction. The mean wrapping efficiency, however, is consistent with the reported crystallographic value for canonical nucleosomes, with a measured value of 147 ± 3 bp (SEM).

**Figure 3:**
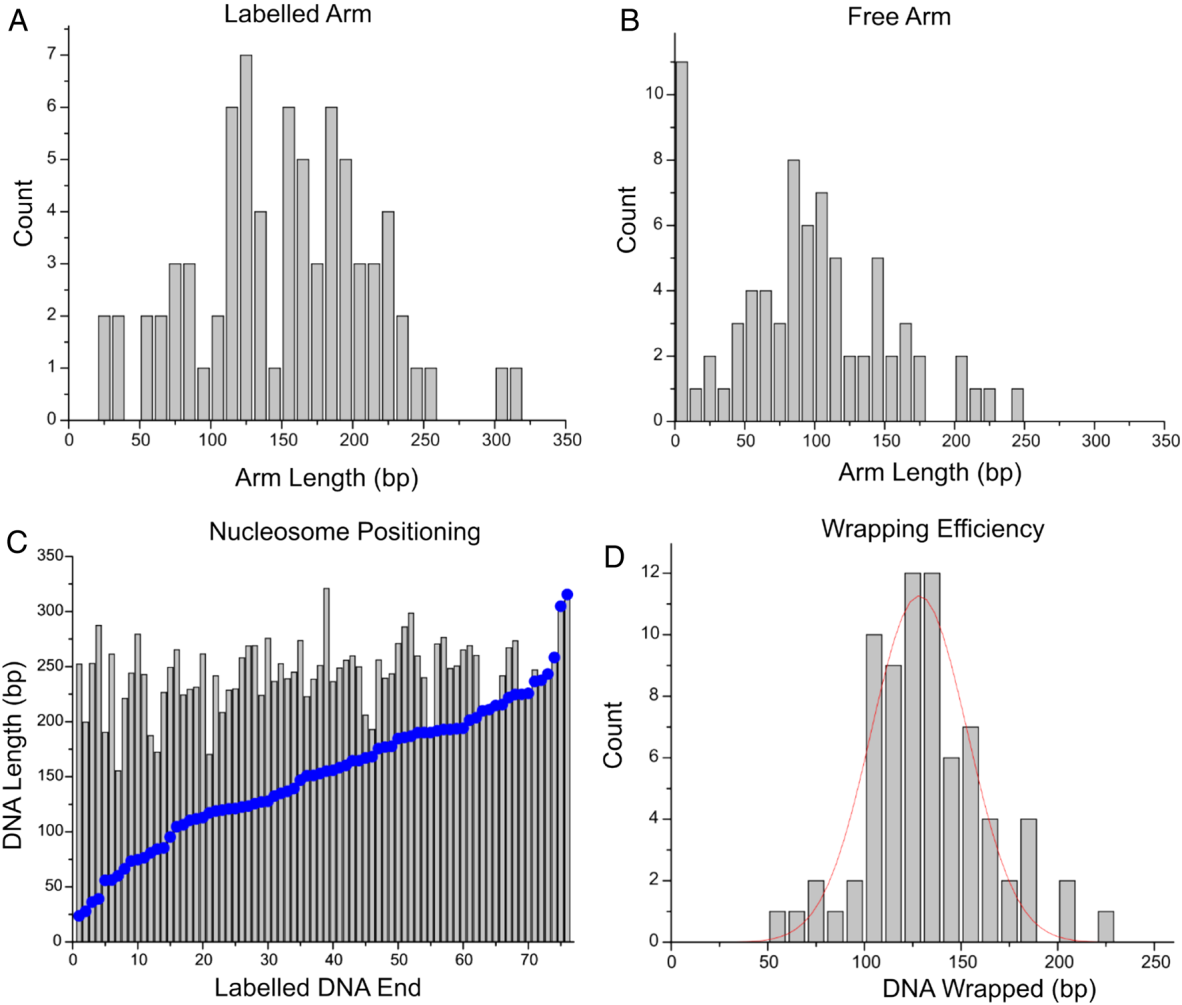
Analysis of modified mononucleosomes with the H4 histone tail truncated assembled on the mixed substrate. Histograms (**A**,**B**) with a bin size of 10 bp of the labelled and free arms do not show strong preferential lengths. A population positioned at the end of the free arm is noted. (**C**) Positional mapping of modified nucleosomes on the positioning substrate demonstrates a lack of positioning preference on a substrate lacking a positioning motif. (**D**) Histogram of nucleosome wrapping efficiency with bin size of 10 bp. The histogram reveals a mean wrapping efficiency of 133 ± 4 bp (SEM), indicating an under wrapped state.

Similar measurements were performed on H4 truncated nucleosomes assembled on the MS substrate and the data is shown in Fig. 3. Flank lengths were first determined by the same method of measuring from the streptavidin labeled or free DNA ends to the center of the nucleosome and subtracting 5 nm. Flank length measurements plotted as histograms in Fig. 3A,B demonstrate that there is no strongly preferred flank length compared to nucleosomes assembled on the 601 positioning sequence (Fig. 2A,B).

The nucleosome positions were mapped in the same manner as they were on the 601 substrate and the results can be seen in Fig. 3C. Nucleosome positions indicated by the blue circles are organized from shortest to furthest distance from the streptavidin-bound end and reveal no positional preference along the substrate, other than a small population positioned at the free DNA end. The wrapping efficiency data for these nucleosomes shown in Fig. 3D demonstrate a consistently under wrapped state, however, with a mean value of wrapped DNA at 133 ± 4 bp (SEM).

### AFM analysis of dinucleosomes with two positioning sequences

**Figure 4:**
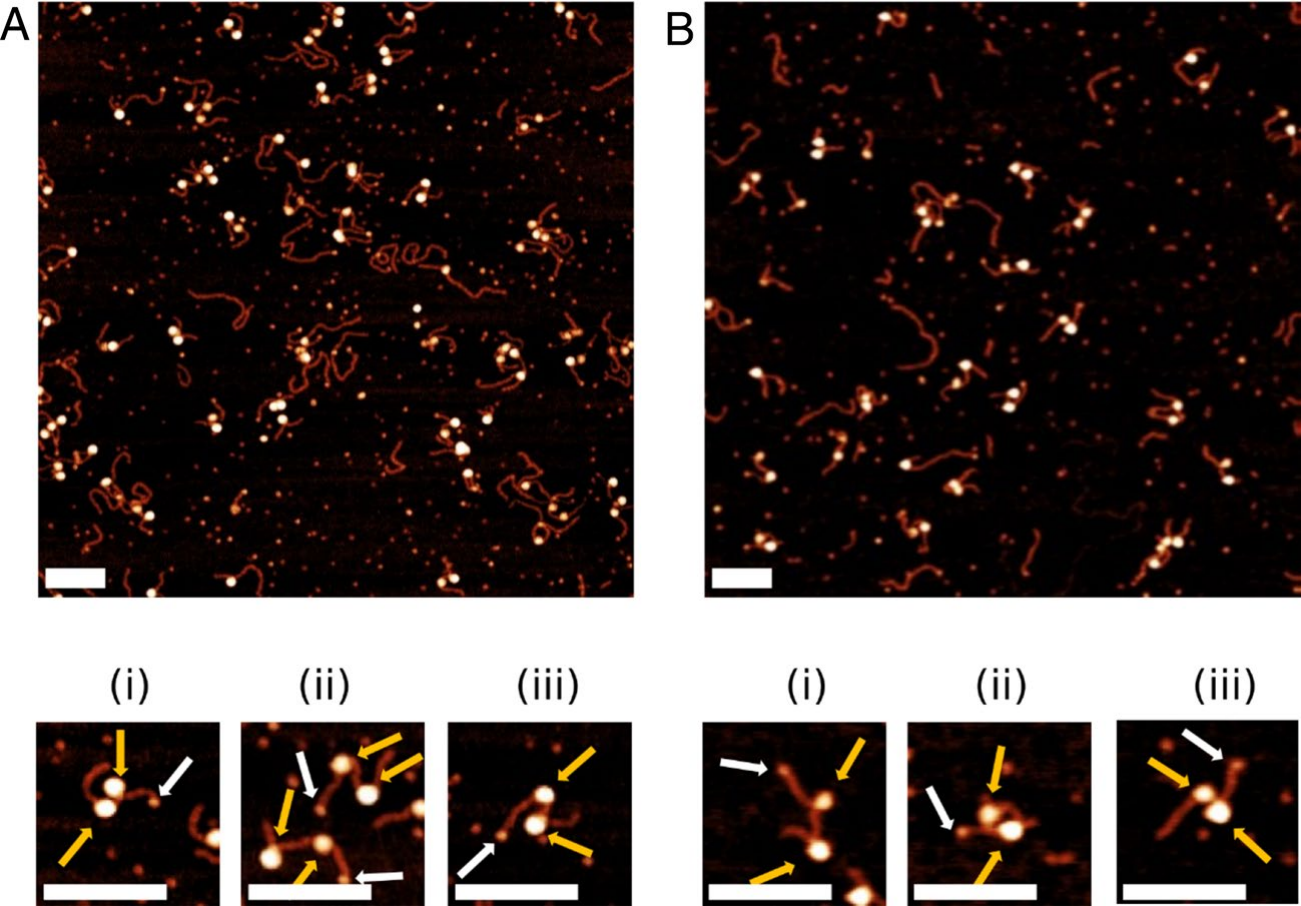
Representative images of dinucleosome samples assembled on the double positioning sequence with snapshots below. White arrows indicate rhizavidin and gold arrows indicate the nucleosome core. Scale bars indicate 100 nm. (i), (ii), and (iii) show nucleosomes with different internucleosomal distances. (**A**) Canonical dinucleosomes. (**B**) H4 truncated dinucleosomes.

To further explore the role of the H4 tail in chromatin structure and accessibility, we studied its role in the assembly of dinucleosomes. We first assembled dinucleosomes on a template containing two copies of the Widom 601 sequence with a 60 bp linker between them (Fig. S1C). Studies of dinucleosomes with two positioning sequences were performed by assembling both canonical nucleosomes and H4 truncated nucleosomes for comparison using the DNA template containing two copies of the Widom 601 sequence. Representative images of assembled dinucleosomes can be seen in Fig. 4, with canonical nucleosomes shown in (A) and H4 truncated nucleosomes shown in (B). Selected complexes are shown below large images. The zoomed snapshots that were selected ((i–iii)) show dinucleosomes assembled with different internucleosomal distances. Gold arrows point to nucleosome core particles and white arrows point to bound rhizavidin. From these images, we obtained characterizing data such as flank lengths, internucleosomal distance, nucleosome position, and mean wrapping efficiency.

The free DNA and flank lengths were determined the same way as for mononucleosomes. Internucleosomal distance, or linker length, was measured as the distance from the center of one nucleosome to the center of the other nucleosome and then subtracted by 10 nm to account for the contribution of the nucleosome core diameter. Rhizavidin was used as a reference point in place of streptavidin on all dinucleosome constructs, as the smaller size of the protein makes the distinction of the reference point clearer in this more complicated system. From the image analysis, parameters such as arm length, wrapping efficiency, internucleosomal distance, and positioning were determined.

Fig. S2 depicts histograms of the measured flank lengths for non-biotinylated (A) and biotinylated (B) flanks of canonical dinucleosomes assembled on this substrate. The histograms for the free and labelled arms have peaks centered at 111 +/− 1 and 114 +/− 1 bp (SEM), respectively, which corresponds to the placement of the two copies of the 601 sequence seen in Fig. S1C. The measured linker lengths of these complexes are shown as a histogram in Fig. S2C with a peak centered at 60 +/− 2 bp (SEM), which is in agreement with the expected linker length of 60 bp.

**Figure 5:**
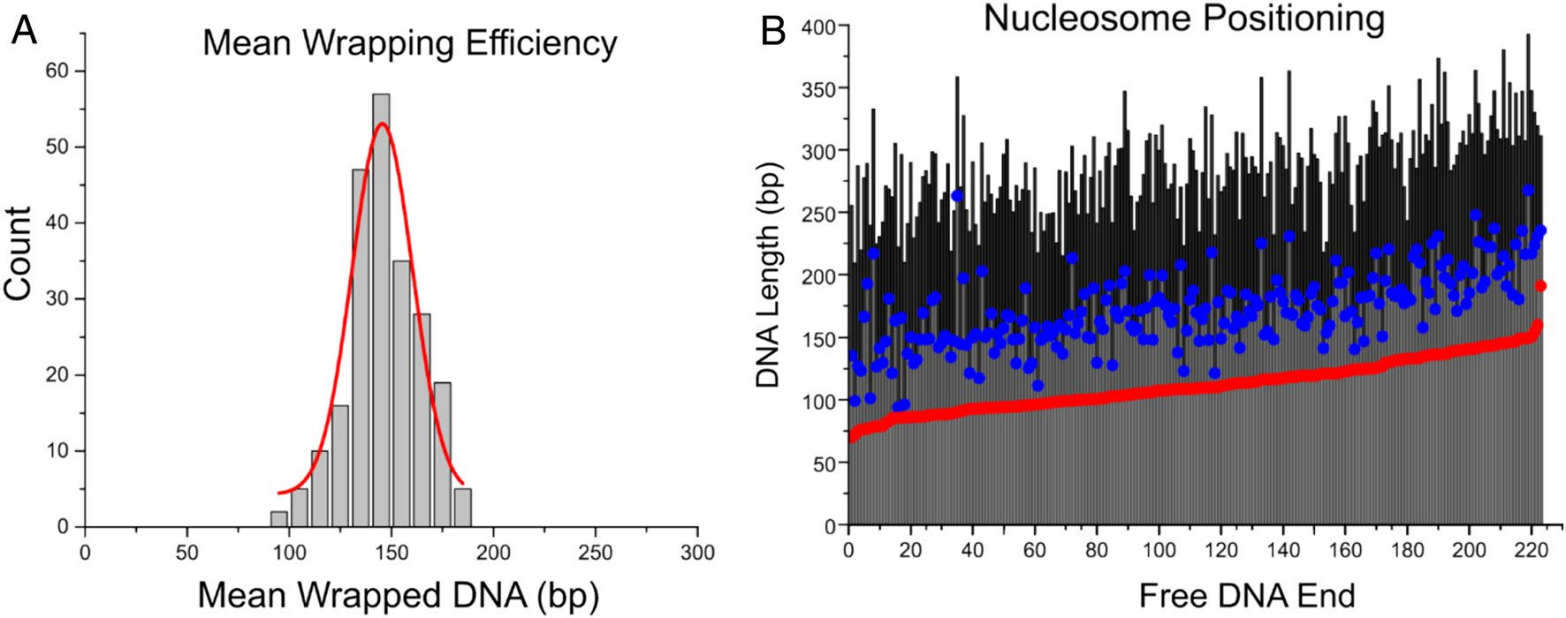
Mean wrapping efficiency and positional mapping of canonical dinucleosomes assembled on the substrate with two positioning motifs. Histogram (**A**) mean value of 146 ± 1 bp (SEM), which closely corresponds to the canonical value of 147 bp. (**B**) Positional mapping of canonical dinucleosomes demonstrates preferential positioning for both nucleosomes.

We measured mean wrapping efficiency for the dinucleosomes in a manner similar to mononucleosomes. We subtracted the measured flank lengths and linker lengths from the known length of the substrate, 578 bp, and divided the result by two. This calculation gives us the mean length of wrapped DNA for both nucleosome cores on the substrate. The histogram representative of these data can be seen in Fig. 5A. The graph shows a narrow peak in mean wrapping efficiency with a mean value of 146 +/− 1 bp (SEM) which coincides with the expected value of 147 bp for canonical nucleosome wrapping.

The canonical nucleosomal positioning data was then mapped and can be seen in Fig. 5B. Dinucleosomes were arranged in order of the position of the first nucleosome, represented by a red circle, measured from the free DNA end. The second nucleosome, represented by a blue circle, was positioned at the distance of the combined free DNA end and linker length. Mapping of the dinucleosomes shows the preferential positioning about the 601 motifs described in Fig. S2. Nucleosomes, with few exceptions, are not positioned randomly on the substrate; rather, they are strongly positioned in the region of the 601 motifs.

**Figure 6:**
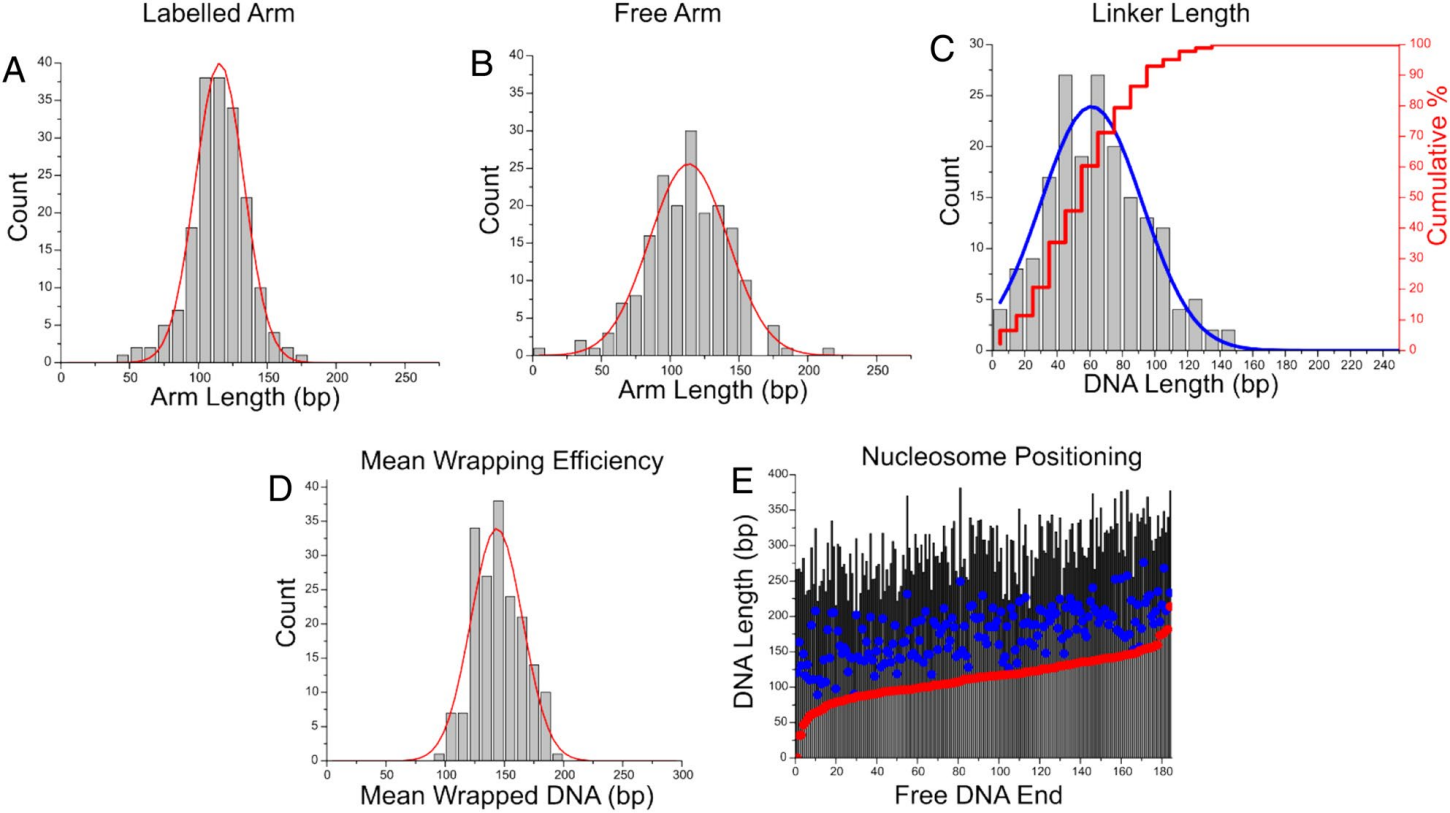
Analysis of modified dinucleosomes with the H4 histone tail truncated assembled on the dinucleosome substrate with two positioning motifs. Histograms (**A**,**B**) reveal mean labelled and free arm lengths of 115 ± 2 bp (SEM) and 113 ± 2 bp (SEM), respectively, which correlate to the position of the 601 motifs. (**C**) Histogram of the linker length between two nucleosomes. The mean linker length value is 65 ± 2 bp (SEM), which is slightly higher and broader than observed for canonical nucleosomes on the same substrate. Histogram (**D**) reveals a mean value of 145 ± 1 bp (SEM). (**E**) Positional mapping of modified dinucleosomes demonstrates a weaker preferential positioning for both nucleosomes when compared with canonical dinucleosomes assembled on the same substrate.

The same analyses performed on canonical dinucleosomes were repeated with H4 truncated dinucleosomes on the same substrate. The histograms of measured arm lengths and linker lengths are shown in Fig. 6. Mean values of these measurements closely resemble that of canonical dinucleosomes; however, the range of linker lengths (Fig. 6C) is distinctly broader than that seen in canonical nucleosomes (31 bp S.D. and 23 bp S.D., respectively). A statistical comparison between overall linker length distribution for canonical nucleosomes and modified nucleosomes with H4 tail truncated assembled on the substrate containing two 601 motifs was performed. Comparison reveals that the distributions are significantly different, at a *P* < 0.01.

Analysis of mean wrapping efficiency in Fig. 6D shows a mean value of 145 +/− 1 bp (SEM). These data coincide with the canonical wrapping value of 147 bp. but the histogram does not reveal the under and over wrapped populations observed in the histograms of H4 truncated mononucleosomes. Mapping was performed in the same fashion as with canonical dinucleosomes and is shown in Fig. 6E. Red circles indicate the first nucleosome on the substrate measured from the free DNA end, and blue circles indicate the second nucleosome. This illustrates that the position of the second nucleosome varies, which is in line with the broad distribution of internucleosomal distance shown in Fig. 6C.

### AFM analysis of dinucleosomes with one positioning sequence

Next, we wanted to understand how truncation of the H4 tail affects internucleosomal interaction of nucleosomes assembled on the substrate containing both 601 and non-specific MS DNA. To address this question, we assembled H4 truncated dinucleosomes in the presence of a single positioning sequence on the template shown in Fig. S1D**.** This template was designed to position one nucleosome while allowing another to freely interact with the DNA. Nucleosomes were assembled, labeled with rhizavidin, imaged, and analyzed in the same fashion as described above and in the Methods section. A representative image of assembled dinucleosomes with zoomed in snapshots of selected complexes can be seen in Fig. S3. Gold arrows point to nucleosome core particles and white arrows point to bound rhizavidin.

**Figure 7:**
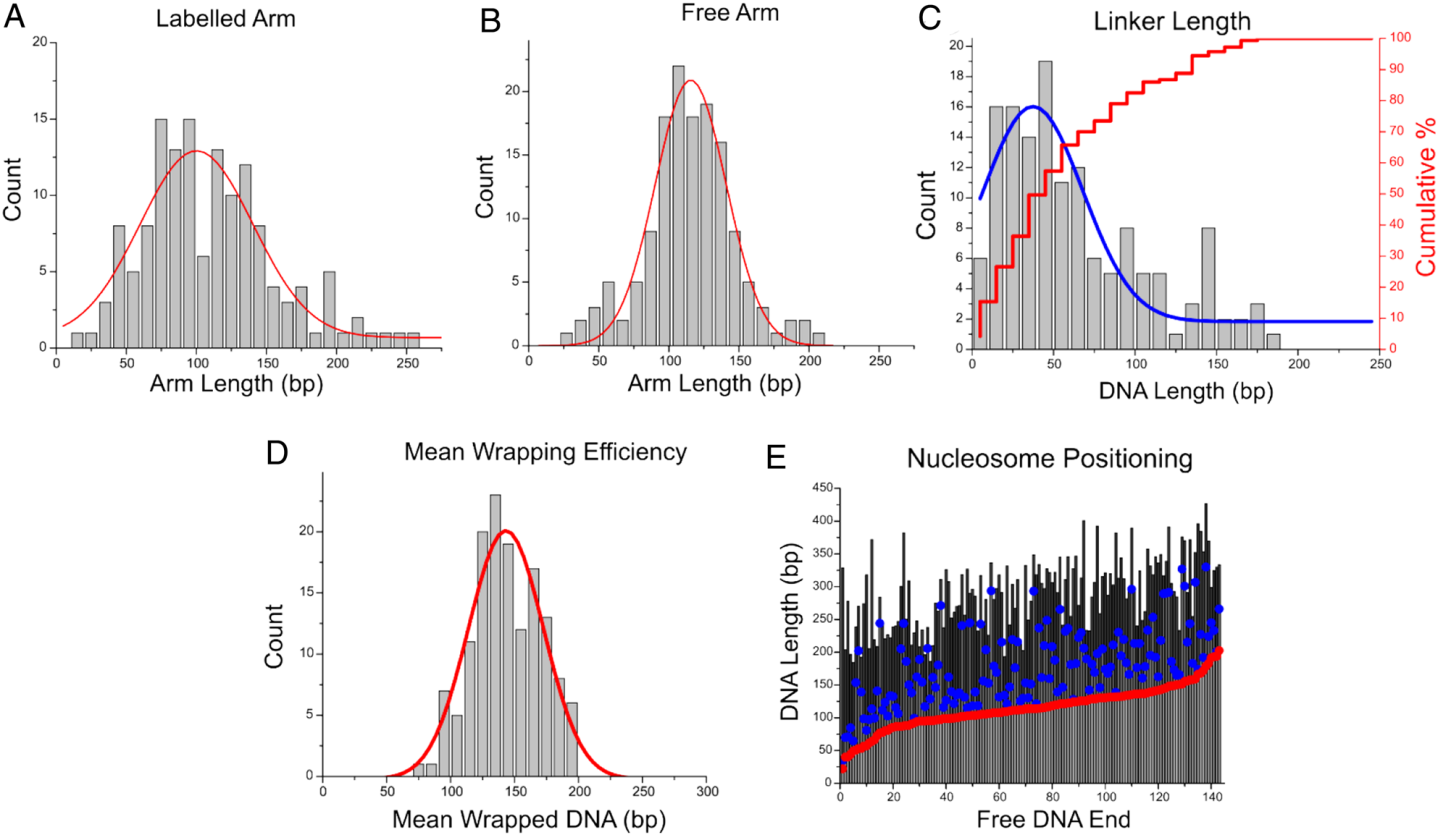
Analysis of modified dinucleosomes with the H4 histone tail truncated assembled on the dinucleosome substrate with one positioning motif. Histograms (**A**,**B**) depict the labelled and free arm lengths, respectively. The labelled arm shows a broad distribution with mean value of 112 ± 4 bp (SEM), while the free arm has a mean value of 115 ± 3 bp (SEM). Note that the 601 motif is positioned at the end of the free arm. (**C**) Histogram of the linker length between two nucleosomes. Mean linker length value is measured as 64 ± 4 bp (SEM), while a Gaussian fit shows a peak centered at 37 ± 30 bp (SD). Histogram (**D**) reveals a mean wrapping efficiency of 144 ± 2 bp (SEM), indicative of fully wrapped nucleosomes. (**E**) Positional mapping of modified dinucleosomes demonstrates a preference for nucleosomes to be proximal to one another.

Arm and linker length measurements (Fig. 7A,B) reveal a marked difference in this assembly compared with the other nucleosome assemblies in this study. The biotinylated arm (MS DNA, Fig. 7A) demonstrates a broad distribution with a mean value of 115 ± 2 bp (SEM). The non-biotinylated arm (601 DNA, Fig. 7B) shows mean value of 113 ± 2 bp (SEM) and corresponds to the position of the 601 sequence. On this substrate, nucleosomes are not observed to position at the DNA ends. This is a departure from the end-positioning observed for mononucleosomes on the MS substrate.

Analysis of the linker lengths between nucleosomes is shown in Fig. 7C. The peak Gaussian of the histogram is depicted as the blue line and the cumulative percentage of linker lengths at or below a given value is shown by the red stepwise line. While the mean value of linker lengths between nucleosomes is found to be 64 ± 4 bp (SEM), the peak of the Gaussian corresponds to the internucleosomal distance as small as 33 ± 30 bp (SD). Cumulative percentages reveal that more than 50% of all nucleosomes are within 50 bp of one another. This value is considerably greater than the percentages observed for truncated dinucleosomes assembled with two positioning sequences (35%, which corresponds to a 46% increase) and for canonical dinucleosomes assembled with two positioning sequences (30%, which corresponds to a 59% increase).

To determine whether the increase in proximally positioned nucleosomes is caused by the truncation of the H4 tail, we compared our linker length data with that from our previous publication on canonical nucleosomes. Analysis of linker lengths between canonical nucleosomes, modified from our previous publication^26^, is shown in Fig. S4. The mean value of linker lengths is found to be 73 ± 4 bp (SEM), an increase from that seen in the tail-truncated population; however, a statistical comparison between overall linker length distribution for canonical nucleosomes and tail-truncated nucleosomes on this substrate reveals no significant difference, indicating that the populations behave similarly.

We performed analysis of mean wrapping efficiency and dinucleosome mapping of this assembly (Fig. 7). The mean wrapping efficiency is shown in Fig. 7D. The peak is centered at 144 ± 2 bp (SEM), indicating an elimination of the unwrapping effect seen in mononucleosomes. Dinucleosome position mapping was performed in the same manner as before and as described in the methods, and the results are shown in Fig. 7E. The red circle indicates the nucleosome nearest the non-biotinylated DNA end and is typically found positioned on the 601 sequence. The blue circle indicates the nucleosome nearest the biotinylated DNA end and is typically positioned in the MS region. Qualitatively, this map shows preference for neighboring nucleosomes to position proximal to one another, as the nucleosomes in the MS region (blue dots) are often seen to be positioned close to the nucleosomes in the 601 region (red dots). This proximity is represented quantitatively in the linker length measurements (Fig. 7C). Taken together, these data show that the DNA sequence has a strong effect on the internucleosomal interactions allowing for nucleosomes to position near one another.

## Discussion

In this study we revealed the role of the histone H4 tail in nucleosome structure and internucleosomal interaction with the use of AFM. We found that truncation of the H4 tail changes the wrapping efficiency of nucleosomes. This effect is mitigated by the use of a high-affinity positioning sequence or the presence of a second nucleosome. Importantly, H4 truncation does not diminish the frequency of internucleosomal interaction. Below we discuss these features of the nucleosomes in more detail.

Nucleosomes assembled with tail-truncated H4 histones displayed a marked difference in wrapping efficiency distribution when compared with the accepted canonical value of 147 bp. According to Fig. 2D, a minor population of nucleosomes assembled on the Widom 601 sequence exhibit a wrapping efficiency considerably lower than that seen in canonical nucleosomes, with a Gaussian fitted peak of 108 ± 9 bp (SD). This low peak value is indicative of nearly a half-turn unwrapping of the nucleosome complex. However, the nucleosomes still position faithfully to the 601 region of the DNA substrate. In the case of mononucleosomes assembled on the MS DNA, the major population exhibited a decreased wrapping efficiency of 133 ± 4 bp (SEM, Fig. 3D), while positioning along the substrate was effectively random. These data suggest that the H4 histone tail plays a critical role in the nucleosome-DNA interaction that defines its degree of wrapping and stability, as the nucleosome structure is altered when the H4 tail is truncated. This unwrapping results in a more open nucleosome conformation, which can be associated with more transcriptionally active regions in chromatin. Moreover, histone H4 cleavage and subsequent opening of chromatin by protease granzyme A has been shown to induce apoptosis^27^. Therefore, the H4 tail is implicated in regulation of nucleosome structure, and on a larger scale, regulation of gene expression and cellular function. Additionally, the DNA sequence itself is shown to be important in regulating the structure of the nucleosome- the highly specific Widom 601 sequence is capable of largely maintaining a fully wrapped conformation of the nucleosome in the absence of the H4 tail, whereas the MS DNA is unable to do so, resulting in altered nucleosome structure. Altogether, these results suggest that nucleosome wrapping efficiency and stability are dependent both on DNA sequence and histone tail-DNA interaction.

The results for dinucleosomes reveal more interesting features of the tail-truncated nucleosomes. When looking at the mean wrapping efficiency data for the H4 tail-truncated nucleosomes assembled on the substrate containing two positioning sequence (Fig. 6D), we see that the nucleosomes adopt a fully wrapped conformation, compared with the under wrapped populations observed in mononucleosome samples. This points to a possible enhanced internucleosomal interaction stabilizing the nucleosomes structures in the H4 tail-truncated dinucleosomes. Moreover, while the mean internucleosomal distance is comparable with that of canonical nucleosomes assembled on the same substrate (Fig. 6C), the distribution is markedly broader than that observed with canonical dinucleosomes, resulting in a significant difference between populations (*P* < 0.01). Internucleosomal interactions that emerge appear to strengthen the stability of each nucleosome complex. On the other hand, the increased range of internucleosomal distance indicates a weakened histone-DNA interaction for the tail- truncated H4, allowing the nucleosome to position more freely along the DNA sequence. These results suggest a complex interplay between internucleosomal interaction and histone-DNA interaction.

Looking at the internucleosomal interaction further, H4 tail-truncated dinucleosomes assembled on the substrate containing a single positioning sequence provide further insight into the effect of the H4 tail. Figure 7C reveals the internucleosomal distance of these nucleosomes. We see that the distance is effectively halved in the major population by using only a single positioning sequence. This indicates that when one nucleosome is located at the positioning sequence and the second one is freely able to interact with the DNA, the nucleosomes interact more closely with one another, even in the absence of the H4 tail.

Therefore, we suggest the H4 tail plays an important role in stabilizing histone-DNA interactions, rather than internucleosomal interactions. Moreover, the interactions between nucleosomes are able to overcome any loss of stability induced by the truncation of the H4 tail. With the elimination of the H4 tail, it is easier to observe the effects that the H3, H2A and H2B tails might be contributing to the stability of nucleosomes^17^. The H4 tail has been shown previously to bind to the acidic patch of the H2A/H2B of a neighboring nucleosome^28^. The H2B N-terminal tail can make contacts with DNA and with the elimination of the H4 tail, these interactions become more important in stabilizing adjacent nucleosomes^29^. It is likely that the increase in stability seen in the dinucleosomes constructs, that is not seen in the mononucleosome construct, is due to the remaining tails interacting with the neighboring nucleosome.

As discussed above, the H4 tail is known to interact with adjacent nucleosomes via contact with the H2A/H2B acidic patch of the neighboring nucleosome. This interaction is thought to stabilize the structure and regulate nucleosomal spacing. Mean wrapping efficiency data for dinucleosomes revealed populations of both under and over wrapped nucleosomes. We looked more closely at this phenomenon on the substrate containing two positioning sequences with both canonical and H4 tail-truncated dinucleosomes. A detailed analysis was performed to compare and understand the relationship between mean wrapping efficiency and internucleosomal distance. To explore this, we separated the dinucleosomes into three categories- those in an under wrapped state (< 130 bp mean wrapping efficiency), a normally wrapped state (130–160 bp), and an over wrapped state (> 160 bp) and measured their position and internucleosomal distances. Fig. S5 shows the results. When two positioning sequences are used, the linker lengths are similar in all populations. Under wrapped canonical dinucleosomes show a mean linker length of 91 ± 5 compared with 80 ± 4 bp (SEM) for tail-truncated dinucleosomes. Normally wrapped nucleosomes exhibit a mean linker length of 61 ± 2 and 69 ± 3 bp (SEM) for canonical and tail-truncated dinucleosomes, respectively. The over wrapped populations reveal mean linker lengths of 42 ± 2 and 41 ± 3 bp (SEM) for canonical and tail-truncated dinucleosomes, respectively. This data indicates a direct relationship between increased wrapping efficiency and reduced linker length. This relationship exists in both the presence and absence of the histone H4 tail.

We performed the same analysis on the H4 tail-truncated nucleosomes assembled on the substrate containing only one positioning sequence (Fig. S6). The under wrapped population has a wide range of observed internucleosomal distances with no obvious preference. Close contact between nucleosomes is rare- only 38% of under wrapped dinucleosomes were positioned within 50 bp of one another. This indicates that, when nucleosomes are furthest apart and internucleosomal interaction is weak or absent, the nucleosomes adopt the more open state observed in mononucleosomes. The over wrapped population, on the other hand, was commonly positioned near one another, despite the lack of a second positioning sequence- 72% of all of all over wrapped dinucleosomes were positioned within 50 bp of one another. As nucleosomes approach one another, the internucleosomal interaction can be enhanced and result in a higher degree of wrapping and stability of the nucleosome, even when the H4 tail is truncated. These results support the idea that internucleosomal interactions, not histone-DNA interactions, are the most important factor in chromatin compaction.

## Methods

### DNA substrates

The DNA substrates used in nucleosome assembly were generated using PCR with a pUC57 plasmid vector from BioBasic (Markham, ON, CA). A biotinylated reverse primer (IDT, Coralville, IA) was used on all substrates for streptavidin or rhizavidin labeling.

For the mononucleosome substrates, two constructs were used; one construct featuring 147 bp of the strong positioning Widom 601 sequence^30^ flanked by plasmid DNA of 113 and 117 bp, and another substrate which replaces the 147 bp of the Widom 601 sequence with a non-specific sequence. Two dinucleosome substrates were also used; one featured the 147 bp of the Widom 601 sequence flanked by 113 and 117 bp, while the other contained two copies of the Widom 601 sequence, separated by 60 bp and flanked by 110 and 114 bp. The substrate constructions are presented in Fig. S1**.** DNA Sequences are listed as Fig. S7. DNA substrates were separated by gel electrophoresis using 1% SeaKem LE Agarose gel (Lonza Group AG, Basel, Switzerland). The bands were excised and purified using QIAquick Gel Extraction Kit (Qiagen, Hilden, Germany). DNA concentration was then determined using NanoDrop Spectrophotometer ND-1000 (Thermo Fischer, Waltham, MA).

### Nucleosome assembly

Human histone octamers were purchased from The Histone Source (Fort Collins, CO). Compared to the Pubmed H3 sequence, the histone constructs from The Histone Source contain the mutations G102A and G111A. The H4 truncated histones feature a 19 amino acid n-terminal deletion. The sequence is MKVLRDNIQGITKPAIRRLARRGGVKRISGLIYEETRGVLKVFLENVIRDAVTYTEHAKRKTVTAMDVVYALKRQGRTLYGFGG. Nucleosomes were assembled using the stepwise dilution assembly protocol described in papers^26,31,32^. Briefly, recombinant histone octamers were initially dialyzed against dialysis buffer (20 mM Tris pH 7.5, 2 M NaCl, 1 mM EDTA, 5 mM 2-mercaptoethanol) at 4 °C for 1 h. DNA was then mixed with the octamers at a 1:1 molar ratio for mononucleosome samples, while di-nucleosomes were assembled at a molar octamer-to-DNA ratio of 3:1. The mixture was diluted in four steps with incubation for 30 min at each step from an initial buffer concentration of 2 M NaCl to a final buffer concentration of 250 mM NaCl in 10 mM Tris, pH 8.0. NaCl concentrations at each step were 2 M, 1.48 M, 1 M, 600 mM, and 250 mM, respectively. Nucleosomes were then stored at 4 °C. Nucleosome assembly was verified by AFM imaging.

### Labelling of nucleosomes

Nucleosomes were labelled at a terminal biotin using rhizavidin and streptavidin, which bind specifically to the biotinylated DNA terminus. Streptavidin was used for labelling mononucleosomes and rhizavidin^33,34^, a streptavidin variant with smaller size, was used for labelling dinucleosomes. Assembled mononucleosomes were incubated with streptavidin for 5 min at room temperature at a molar ratio of 2:1 streptavidin:nucleosome in incubation buffer (10 mM Tris pH 8.0, 125 mM NaCl, 5 mM MgCl_2_.). Dinucleosomes were incubated with rhizavidin for 5 min at room temperature at a molar ratio of 4:1 rhizavidin:nucleosome in incubation buffer. After incubation, samples were immediately prepared for imaging as described below.

### Atomic force microscopy imaging

Sample preparation for AFM imaging was performed as previously described^26^. Freshly cleaved mica was functionalized with a solution of 1-(3-aminopropyl)- silatrane (APS). The nucleosome stock solution was diluted from 300 to 2 nM in imaging buffer (10 mM HEPES pH 7.5, 4 mM MgCl_2_) immediately before deposition on the functionalized mica. The sample was left to incubate for 2 min before being rinsed with water and dried with argon flow. Samples were stored in vacuum before being imaged on Multimode AFM/Nanoscope IIId system using TESPA probes (Bruker Nano Inc, Camarillo, CA). A typical image captured was 1 × 1 μm in size with 512 pixels/line.

### Data analysis

DNA contour length analysis was performed by measuring from the center of the protein label to the free end of DNA using Femtoscan software (Advanced Technologies Center, Moscow, Russia). Flank measurements for the nucleosomes were obtained by measuring from the center of the protein label to the center of the nucleosome for the labeled arm and from the free end of DNA to the center of the nucleosome for the unlabeled arm. 5 nm was subtracted from each measured flank length to account for the size contributed by the histone core^35,36^. Dinucleosome flanks were measured in the same fashion, while the internucleosomal distance was measured from the center of one nucleosome to the center of the other along the path of the linker DNA. The internucleosomal distance, or linker length, was calculated by subtracting 10 nm from the center-to-center distance values between the two nucleosomes. Free DNA was measured on each image. The mean value of the free DNA was then divided by the known length of a given substrate. The resulting value was used as a conversion unit. All other measurements on each image were divided by the calculated conversion unit to convert measurements in nm to base pairs (bp). Histograms of free DNA measurements used to calculate conversion units can be seen in Fig. S8.

Wrapping efficiency for mononucleosomes was calculated by subtracting the combined flank lengths from the known DNA lengths as it was done before^26,31,32^. Mean wrapping efficiency for dinucleosomes was calculated by subtracting the combined arm and linker lengths from the known DNA lengths and dividing by two. These methods were used to produce histograms and mapping of nucleosome position and wrapping efficiency. Subsets of these data were sorted by wrapping efficiency to compare nucleosome structure and interaction at various wrapping states. Data was separated into bins of nucleosomes wrapped less than 130 bp, nucleosomes wrapped 130–160 bp, and nucleosomes wrapped more than 160 bp. All graphs were created using Origin, Version 6.0 (OriginLab Corporation, Northampton, MA, USA). Gaussian fitting was performed automatically using Origin software’s “Fit Gaussian” analysis function on each histogram. The reported peak values correspond to the output of the “Fit Gaussian” analysis function. A non-parametric Kolmogorov Smirnov method was used to determine the statistical significance of linker length distribution and subpopulations of linker lengths based on wrapping efficiency between different dinucleosome substrates.

## Supplementary Information

Supplementary Information.

## Data Availability

The datasets generated during and/or analyzed during the current study are available from Y.L.L. (ylyubchenko@unmc.edu) upon reasonable request.
