# Supplementary material for: Effect of histone H4 tail on nucleosome stability and internucleosomal interactions
Source: Sci Rep. 2021 Dec 16;11:24086. doi: 10.1038/s41598-021-03561-9 (PMC8677776; doi:10.1038/s41598-021-03561-9)
Supplement: Supplementary file 1 — Supplementary Information. [file 41598_2021_3561_MOESM1_ESM.pdf]

Effect of Histone H4 Tail on Nucleosome Stability and Internucleosomal Interactions

Supplementary Information

Tommy Stormberg, Sridhar Vemulapalli, Shaun Filliaux, and Yuri L. Lyubchenko

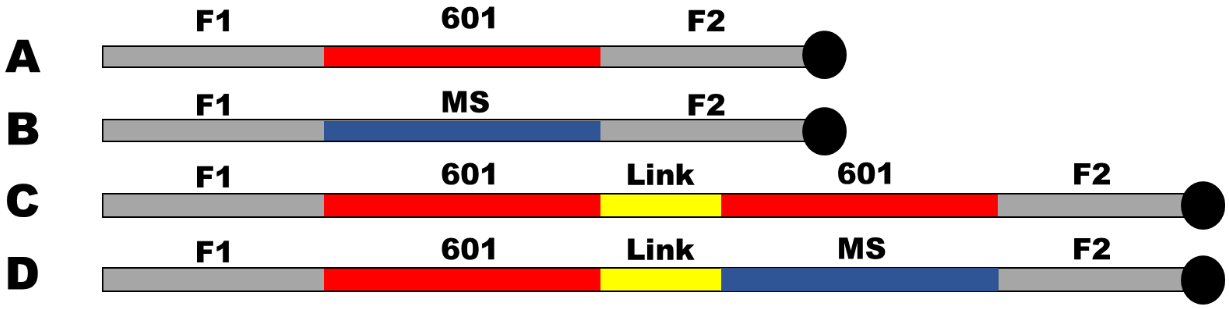

Fig. S1. Schematic representation of the four templates used. Schematics (A) and (B) represent the mononucleosome templates, while (C) and (D) represent the dinucleosome templates. Sequence F1 and F2 represent flanks 1 and 2, composed of nonspecific sequences.F1 is 110 bp and F2 is 114 bp on template (C). On all other templates, F1 is 113 bp and F2 is 117 bp. Sequence 601 represents the Widom 601 sequence of 147 bp. MS represents a non-specific sequence of 147bp. Link represents the linker DNA, a non-specific sequence of 60 bp. The black circles represent the biotin label at the end of each template.

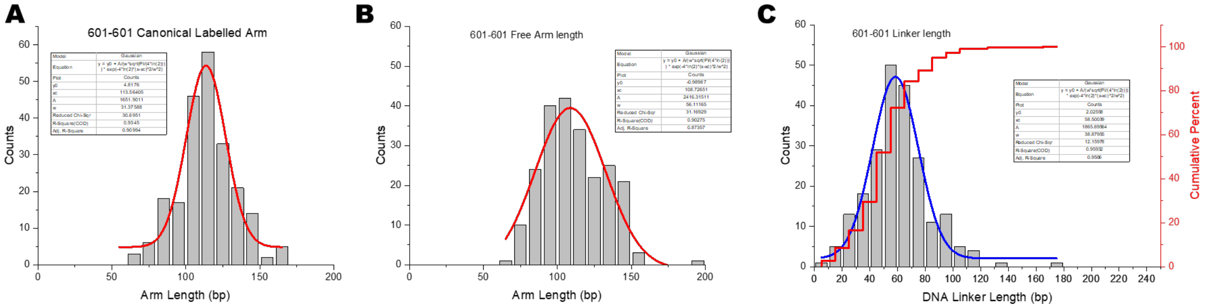

Fig. S2. Analysis of canonical dinucleosomes assembled on the dinucleosome substrate with two positioning motifs. Histograms **(A)** and **(B)** reveal mean values of the labelled and free arm lengths as 114 ± 1 bp (SEM) and 111 ± 1 bp (SEM), respectively, which correlate to the position of the 601 motifs. **(C)** Histogram of the linker length between two nucleosomes. The data reveals a mean linker length of 60 ± 2 bp (SEM), which is in agreement with the distance between positioning motifs (60 bp).

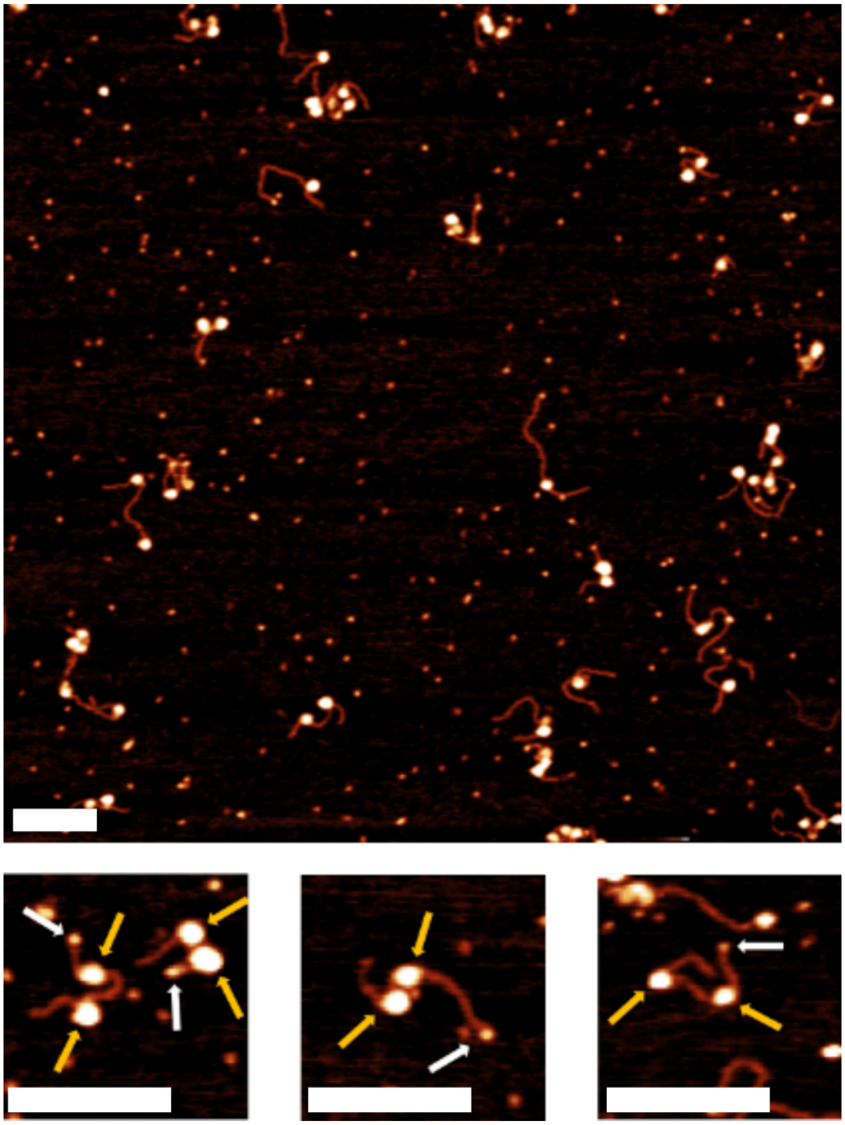

Fig. S3. Representative images of H4 truncated dinucleosome samples assembled on the single positioning sequence with snapshots below. White arrows indicate rhizavidin and gold arrows indicate the nucleosome core. Scale bars indicate 100 nm.

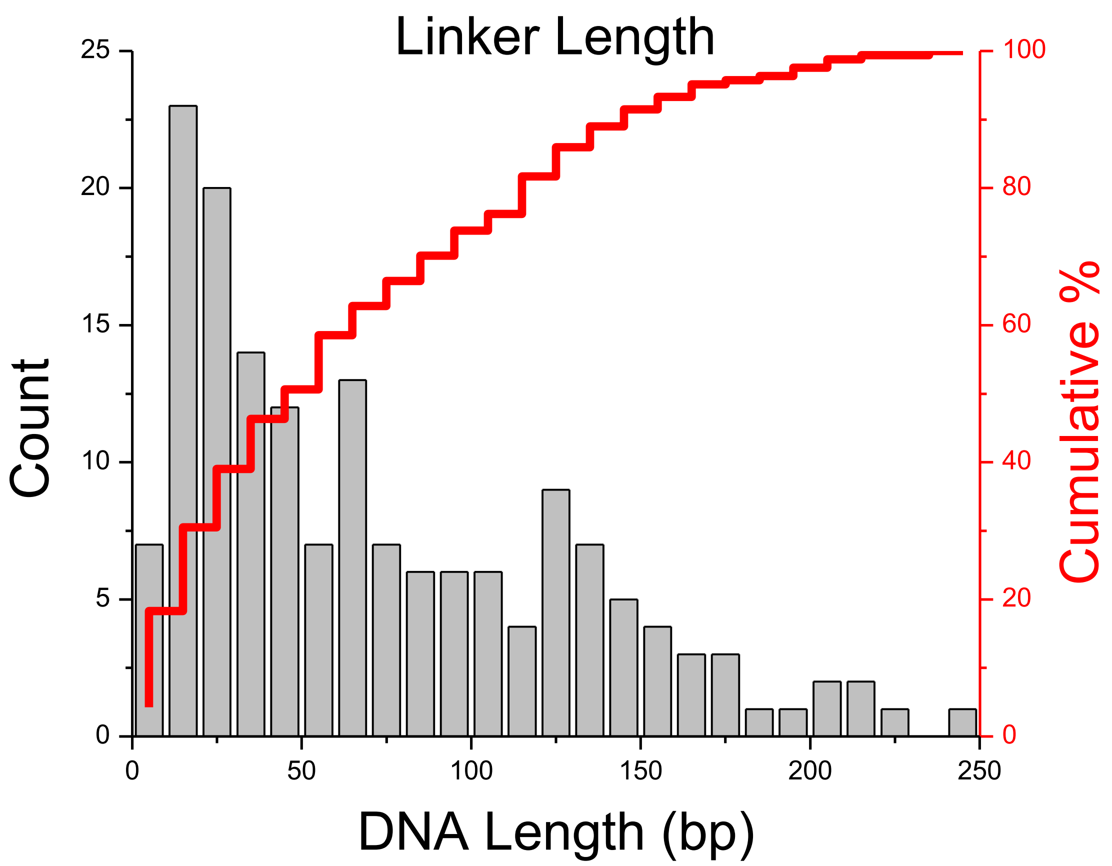

Fig. S4. Histogram of linker lengths for canonical dinucleosomes assembled on the single positioning sequence. Modified from[26].

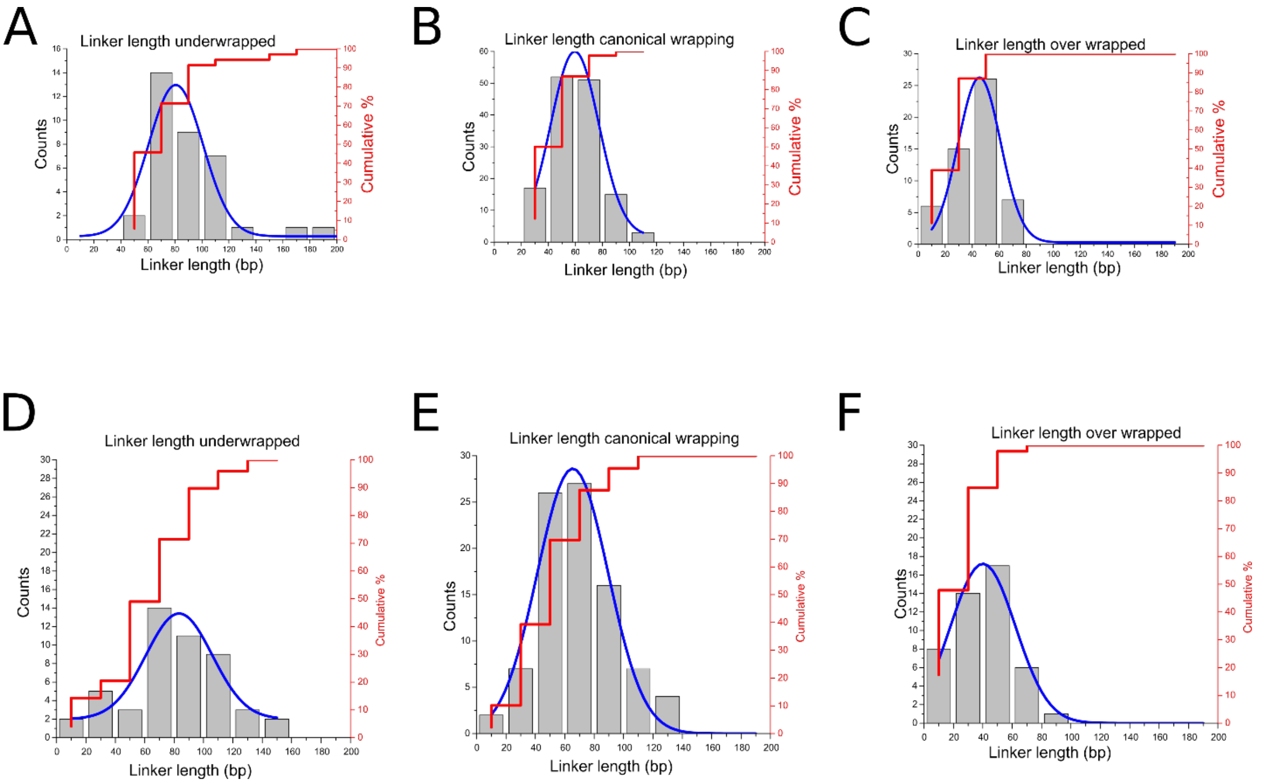

Fig. S5. Histograms of linker length based on mean wrapping efficiency. **(A-C)** depict canonical dinucleosomes and **(D-F)** depict H4 tailless dinucleosomes. Under wrapped conformations correspond to a linker length above the expected length, and over wrapped conformations correspond to a decreased linker length.

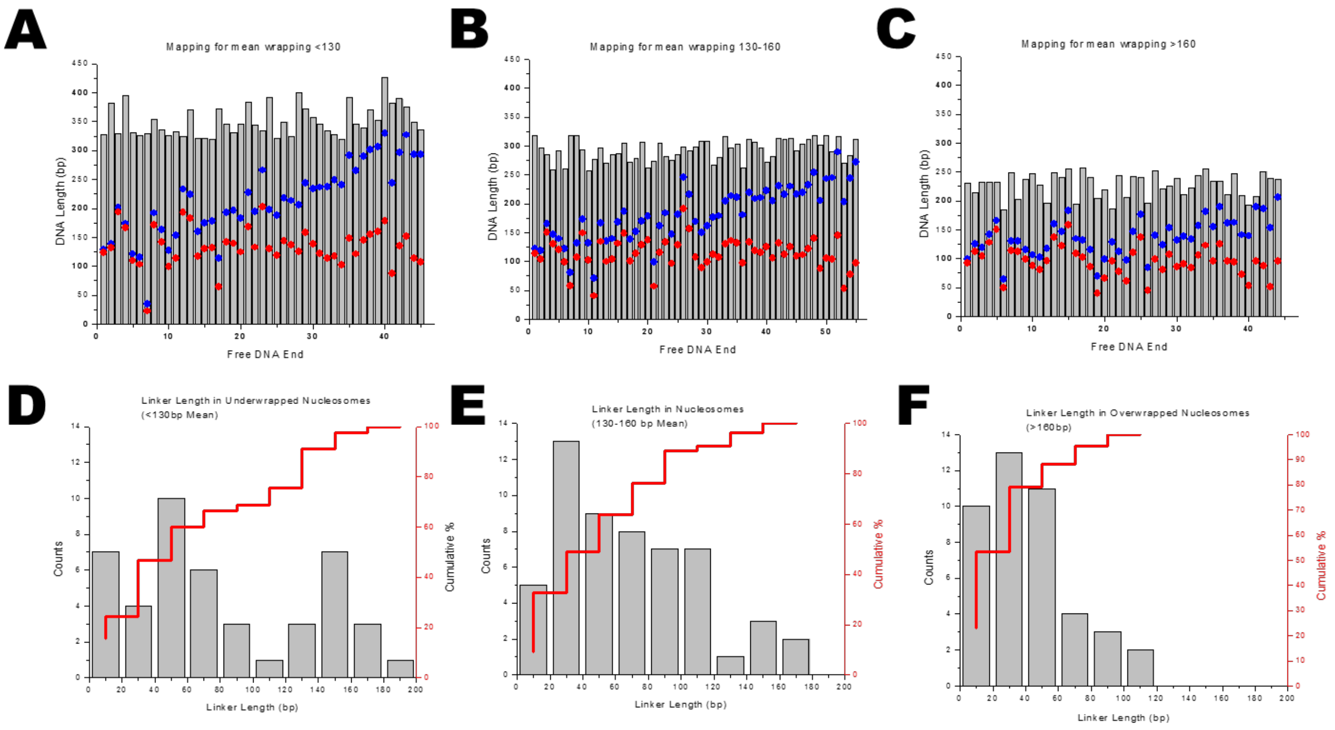

Fig. S6. Analysis of modified nucleosomes assembled on the dinucleosome substrate containing a single positioning sequence according to mean wrapping efficiency.

Sequence A:
5’GATGTGCTGCAAGGCGATTAAGTTGGGTAACGCCAGGGTTTTCCCAGTCACGACGTTGTAAAACGACGGCCAGT
GAATTCGAGCTCGGTACCTCGCGAATGCATCTAGATGACACAGGATGTATATATCTGACACGTGCCTGGAGACTA
GGGAGTAATCCCCTTGGCGGTTAAAACGCGGGGGACAGCGCGTACGTGCGTTTAAGCGGTGCTAGAGCTGTCTA
CGACCAATTGAGCGGCCTCGGCACCGGGATTCTCCAGGTCATCGGATCCCGGGCCCGTCGACTGCAGAGGCCTGC
ATGCAAGCTTGGCGTAATCATGGTCATAGCTGTTTCCTGTGTGAAATTGTTATCCGCTCACAATTCCACACAACATA
CG3’

Sequence B:
5’GATGTGCTGCAAGGCGATTAAGTTGGGTAACGCCAGGGTTTTCCCAGTCACGACGTTGTAAAACGACGGCCAGT
GAATTCGAGCTCGGTACCTCGCGAATGCATCTAGATGACCCTGGGGTGCCTAATGAGTGAGCTAACTCACATTAAT
TGCGTTGCGCTCACTGCCCGCTTTCCAGTCGGGAAACCTGTCGTGCCAGCTGCATTAATGAATCGGCCAACGCGCG
GGGAGAGGCGGTTTGCGTATTGGGCGCTCTTCCGGACATCGGATCCCGGGCCCGTCGACTGCAGAGGCCTGCAT
GCAAGCTTGGCGTAATCATGGTCATAGCTGTTTCCTGTGTGAAATTGTTATCCGCTCACAATTCCACACAACATACG
3’

Sequence C:
5’GATGTGCTGCAAGGCGATTAAGTTGGGTAACGCCAGGGTTTTCCCAGTCACGACGTTGTAAAACGACGGCCAGT
GAATTCGAGCTCGGTACCTCGCGAATGCATCTAGATACAGGATGTATATATCTGACACGTGCCTGGAGACTAGGG
AGTAATCCCCTTGGCGGTTAAAACGCGGGGGACAGCGCGTACGTGCGTTTAAGCGGTGCTAGAGCTGTCTACGAC
CAATTGAGCGGCCTCGGCACCGGGATTCTCCAGGGCGGCCGCGTATAGGGTCCATCACATAAGGGATGAACTCG
GTGTGAAGAATCATGCTTTACAGGATGTATATATCTGACACGTGCCTGGAGACTAGGGAGTAATCCCCTTGGCGG
TTAAAACGCGGGGGACAGCGCGTACGTGCGTTTAAGCGGTGCTAGAGCTGTCTACGACCAATTGAGCGGCCTCG
GCACCGGGATTCTCCAGATCGGATCCCGGGCCCGTCGACTGCAGAGGCCTGCATGCAAGCTTGGCGTAATCATGG
TCATAGCTGTTTCCTGTGTGAAATTGTTATCCGCTCACAATTCCACACAACATACG3’

Sequence D:
5’GATGTGCTGCAAGGCGATTAAGTTGGGTAACGCCAGGGTTTTCCCAGTCACGACGTTGTAAAACGACGGCCAGT
GAATTCGAGCTCGGTACCTCGCGAATGCATCTAGATGACACAGGATGTATATATCTGACACGTGCCTGGAGACTA
GGGAGTAATCCCCTTGGCGGTTAAAACGCGGGGGACAGCGCGTACGTGCGTTTAAGCGGTGCTAGAGCTGTCTA
CGACCAATTGAGCGGCCTCGGCACCGGGATTCTCCAGGGCGGCCGCGTATAGGGTCCATCACATAAGGGATGAA
CTCGGTGTGAAGAATCATGCTTTCCTGGGGTGCCTAATGAGTGAGCTAACTCACATTAATTGCGTTGCGCTCACTG
CCCGCTTTCCAGTCGGGAAACCTGTCGTGCCAGCTGCATTAATGAATCGGCCAACGCGCGGGGAGAGGCGGTTTG
CGTATTGGGCGCTCTTCCGGACATCGGATCCCGGGCCCGTCGACTGCAGAGGCCTGCATGCAAGCTTGGCGTAAT
CATGGTCATAGCTGTTTCCTGTGTGAAATTGTTATCCGCTCACAATTCCACACAACATACG3’

Fig. S7. DNA sequences used for experiments.

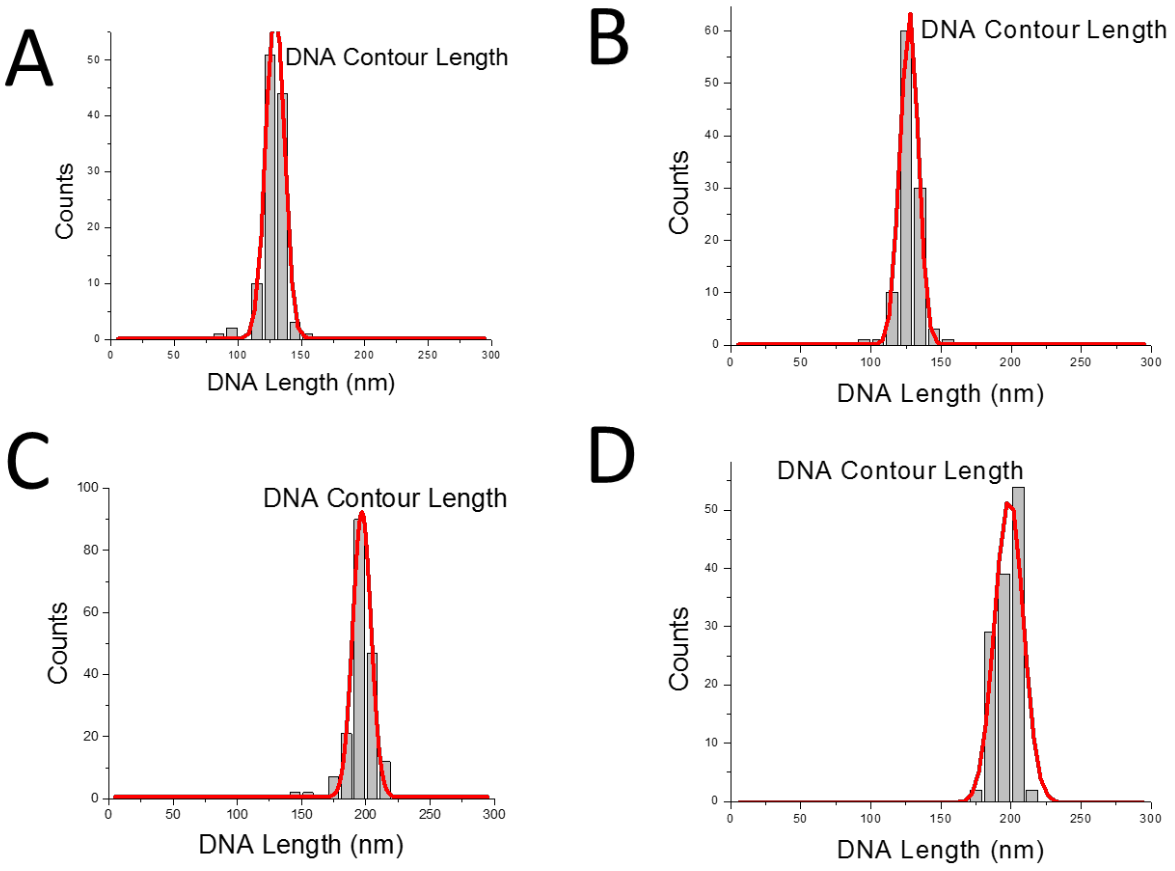

Fig. S8. Histograms of DNA contour length measurements for bp conversions. **(A)** Sequence A DNA. **(B)** Sequence B DNA. **(C)** Sequence C DNA. **(D)** Sequence D DNA.
